# Health seeking behaviour, health system experience and tuberculosis case finding in Gambians with cough

**DOI:** 10.1186/1471-2458-6-143

**Published:** 2006-06-05

**Authors:** Yaya Kasse, Momodou Jasseh, Tumani Corrah, Simon A Donkor, Martin Antonnio, Adama Jallow, Richard A Adegbola, Philip C Hill

**Affiliations:** 1 Tuberculosis Division, Medical Research Council Laboratories, Banjul, The Gambia; 2 National Leprosy and Tuberculosis control Programme, Banjul, The Gambia

## Abstract

**Background:**

Studies in Africa investigating health-seeking behaviour by interviewing tuberculosis patients have revealed patient knowledge issues and significant delays to diagnosis. We aimed to study health-seeking behaviour and experience of those with cough in The Gambia and to identify whether they had tuberculosis.

**Methods:**

During a round of a population under 3-monthly demographic surveillance, we identified people >10 years old who had been coughing ≥ 3 weeks. A questionnaire was administered concerning demographic data, cough, knowledge, health seeking, and experience at health facilities. Case finding utilised sputum smear and chest X-ray.

**Results:**

122/29,871 coughing individuals were identified. Of 115 interviewed, 93 (81%) had sought treatment; 76 (81.7%) from the health system. Those that visited an alternative health provider first were significantly older than those who visited the health system first (p = 0.03). The median time to seek treatment was 2 weeks (range 0 – 106). 54 (58.1%) made their choice of provider because they believed it was right. Of those who left the health system to an alternative provider (n = 13): 7 believed it was the best place, 3 cited cost and 2 failure to improve. 3 cases were identified by sputum analysis, 11 more by X-ray; all had visited the health system first. Total 'excess' cough time was 1079 person weeks.

**Conclusion:**

The majority of people with cough in this population seek appropriate help early. Improved case detection might be achieved through the use of chest X-ray in addition to sputum smear.

## Background

Tuberculosis (TB) causes approximately 2 million deaths per year[1]. 98% occur in low-income countries[2]. Directly observed therapy (DOTS), the main strategy for TB control globally, relies on self-presentation of adults from the community and sputum smear for diagnosis. In certain populations, even in the presence of substantial drug-resistance, it is highly effective at reducing *M. tuberculosis *transmissio[3]. However, in Africa, despite considerable DOTS expansion, the incidence rate is rising by approximately 6% per year[4]. While high rates of HIV infection have contributed to this[5], other factors should be considered, such as the level of understanding of TB in the community, health-seeking behaviour and health system performance.

In The Gambia, which has implemented DOTS and has an HIV seroprevalence of 2%[6], the TB case notification rose from 82/100,000 in 1994 to 140/100,000 in 2004[7]. As in many African countries, there is thought to be considerable under-reporting of TB cases. We engaged the quarterly round of a population under demographic surveillance to identify individuals who were coughing for 3 weeks or more, investigate their health seeking behaviour and related experiences and screen them for TB.

## Methods

### Recruitment

The study was approved by the combined Gambia government/MRC ethics committee and took place on the North bank, the River Gambia, approximately 100 kilometres inland. The Demographic Surveillance System (DSS) monitors 43,548 people in Farafenni town (26,592) and surrounding villages (16,956), recording: births, deaths, nuptial events, and movement in and out of each of over 5500 households. The population is characterised by youthfulness and a high growth rate. Health services are provided by a 155-bed hospital and clinics in larger villages. There are private dispensaries and pharmacies plus traditional health providers. Diagnosis of TB is at the main hospital. Confirmed cases receive free treatment; 61 were treated in 2004, from a source population of approximately 80,000 people in the DSS and surrounding district.

During a 3-monthly DSS round from 17 January to 16 April 2005, the head of each household, or an appropriate substitute, was asked if there was any household member over the age of 10 years, coughing for 3 weeks or more. The person who was identified by the head of household with cough of 3 weeks or more was asked to confirm this and advised to go to the nearest health centre. If they asked why, the field worker explained that a cough of 3 weeks or more is not normal. He/she did not mention any possible causes of a cough.

### Investigation of health seeking behaviour

A semi-structured questionnaire was developed. Questions concerned demographic data (age, sex, occupation, marital status), duration of cough, knowledge of TB, time taken to seek healthcare, type of care sought, assistance available and utilised, quality of the treatment received and associated cost. The questionnaire was administered by trained field workers after informed consent. Participants were divided into those coughing for 3 to 7 weeks and those coughing for 8 weeks and more. The second group were interviewed immediately and the first 6 weeks later.

### Case finding for tuberculosis

After interview, case finding was undertaken. Three sputum samples were collected per person over three days and transported to the Farafenni hospital laboratory. Sputum smears were prepared and stained with Ziel-Neehlsen for acid-fast bacilli. For a positive result, acid-fast bacilli were seen in at least one sputum sample. Each person interviewed was offered a Farafenni hospital chest X-ray, reported by consensus by two specialist physicians, blinded to the sputum results. Those with equivocal X-rays were offered a repeat X-ray after 1 month. Those diagnosed with TB were referred to the National TB control programme.

For quality control, 9 individuals with negative sputum smears and a normal chest X-ray had 2 sputum samples taken to the MRC laboratory. They were prepared and stained with auramine-phenol[8]. positive smears were confirmed by Ziel-Neehlsen (ZN) staining. All were AFB negative. Three sputum smear negative cases each provided 2 repeat sputum samples for smear and culture. Decontaminated specimens were inoculated into BACTEC 9000 MB medium and positive samples were confirmed by ZN stain[9]. All 3 were smear negative but culture positive.

### Data management and analysis

All data were double-entered into an ACCESS database and checked for errors. The data were analysed using STATA (version 8; Stata Corp, College Station, TX). Categorical variables were compared using Chi-Square and quantitative variables were compared using t-test and the sign rank sum test where appropriate.

## Results

The study population from the DSS round was 29,871 over 10 years old. The survey identified 122 individuals (0.4%) coughing for 3 weeks or more; 115 (93.5%) agreed to be interviewed, 8 had travelled, 2 refused, and one had died. Those interviewed were more likely to be villagers, were older than, and had a different ethnic mix to, their source population (table 1). The majority were illiterate (83%), the most common occupation was farming (49%), 63% were married, 19% were single, 14% were widowed and 4% divorced. No significant differences in basic characteristics or health seeking behaviour were found between those interviewed immediately and those interviewed after 6 weeks.

**Figure 1:**
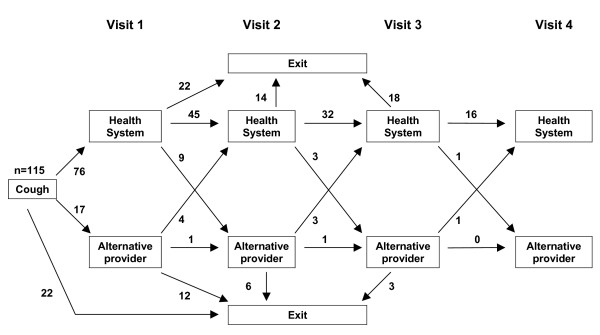
Flow chart of health seeking behaviour of 115 individuals with cough according to attendance at the 'health system' or an alternative provider.

Approximately 80% of interviewees had heard of TB, that it is transmissible and curable (table 2). Radio was the most common source of knowledge. Only 10% knew that a germ caused TB and fewer than 50% could name any key TB signs or symptoms. A large majority (93; 81%; Figure 1) had sought treatment for their cough either at health facilities (hospital, health centres, private physicians) or alternative health services (Pharmacists, traditional healers, drug shop keepers). Only 2 individuals said their decision to seek treatment was influenced by the DSS team. Those who initially went to an alternative provider were significantly older than those who attended the health system first (mean median 55 years, range 30–90 years; versus median 44 years, range 10–80 years; p = 0.03). However there were no significant gender or ethnic differences. The pattern of behaviour of villagers was similar to that of urban residents, except that they were more likely not to seek help at all: 14 (25%) villagers did not seek treatment versus 8 (13%) urban residents (p = 0.1).

Fifty-two (55.9%) participants decided to seek help and where to go by themselves, while a close relative intervened in 25 (26.9%) cases and another compound or village resident in 14 (15.1%) cases. Over half (54; 58.1%) made their choice of provider because they believed it was most appropriate; 19 (20.4%) cited proximity to where they lived and only 4 stated that cost was the primary factor. There were no significant differences in these decision processes between those who initially sought help at the health system and those who first visited an alternative provider, although this analysis was limited by the small sample size. The main reasons given for not seeking treatment were lack of knowledge about the importance of a chronic cough (8) and inability to get to a health centre through lack of resource or transport (10).

The median duration of cough was 10 weeks (mean 37; range 3–572). The median time to seek treatment after the start of coughing was 2 weeks, both for those who visited the health system first (range 0 – 106) and those who visited an alternative provider (range 1 – 52); 81% sought treatment between 1 to 4 weeks. The majority took over half an hour to get to a health provider (51; 67%); the average cost of the trip was 26 Dalasis (1 Euro), for which 7% had to borrow money.

Of those who sought treatment at a health facility, 17 (22%) were asked to provide a sputum specimen for analysis at their first visit, including 9 (19%) of those coughing for less than 3 weeks at the time; one was diagnosed as a case and 3 were later diagnosed through case finding. Eight individuals (18%) who visited a health facility a second time were asked to provide a sputum specimen; all were negative but 3 were later diagnosed as a case in our study. Of those who visited a health facility a third time, 3 (9%) were asked to provide a sputum specimen; all were negative. Two (13%) of those who visited a health facility a fourth time were asked to provide a sputum sample; both were negative. Nine individuals were asked to have a chest x-ray, including 2 who were later diagnosed as a case in our study. None of the X-rays were available for inspection.

The reasons why people left the health system to an alternative provider (n = 13, figure 1) were varied: 7 believed that it might be the best place, 3 cited cost as the main reason and 2 cited failure to improve after treatment. Seventy-five (80.6%) of those who initially sought treatment had stopped seeking help by the interview; 71 gave a reason: 30 (42%) were feeling like they were getting better, 8 were not sure what they were going to do, 7 were still completing a course of treatment and 5 were 'too busy'. However 8 (11%) cited cost as the main reason and 6 (9%) had lost confidence in health providers.

Ninety seven (84 %) participants agreed to produce 3 sputum samples for analysis; 12 refused to participate, 4 had travelled, 1 died, and 1 had been diagnosed with TB. Two were found to be sputum smear positive. Eighty-nine individuals agreed to have a chest X-ray; 2 were pregnant, 4 refused, 1 had died and 1 had been hospitalised. There were 27 individuals with an abnormal X-ray. Thirteen of these (14.6% of all X-rays) were highly suggestive of TB, warranting anti-tuberculosis treatment. The chest-X-rays of four other individuals had infiltrates; they were offered a repeat chest X-ray after 1 month: three agreed and repeat X-rays were normal. Other diagnoses were chronic obstructive airways disease (4), congestive hearth failure (2), old scarring (1), interstitial lung disease (1), pleural effusion (1), and diaphragmatic hernia (1).

**Figure 2:**
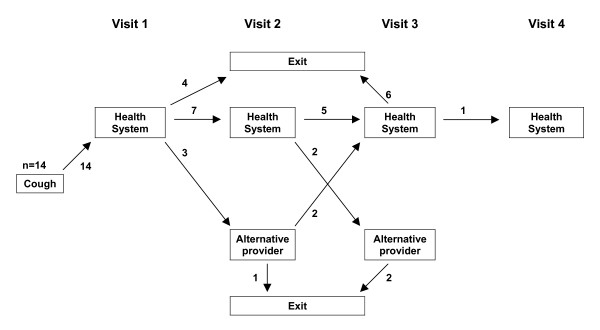
Flow chart of health seeking behaviour of 14 individuals with cough who were diagnosed by the health system (1) or by tracing (13) with TB disease.

The details of the 13 individuals with TB, plus the person who was diagnosed already, are presented in Table 3. The median age of the cases was 57.5 years (mean 55; range 14–80) and almost 2/3 were women. Their health seeking behaviour prior to case finding is shown in figure 2. All 14 visited the health system first. The median time to this visit was 2 weeks (mean 5.7; range 0–52), significantly shorter than the median time to diagnosis (median 21.5; mean 82.8; range 3–572; p = 0.001). The total 'extra' cough time for those eventually diagnosed was 1079 person weeks.

## Discussion

In this study, most coughing individuals sought help from the 'health system' promptly, while 1/3 either sought assistance from an alternative provider or no assistance at all. They were largely guided by a desire to go to the most appropriate place, but after the initial visit health seeking behaviour was complex: those who first sought help within the health system tended to stay in that system, while few people visited an alternative provider more than once and tended to not seek help again. Only a minority of individuals who visited a health facility were asked to provide a sputum sample for analysis and very few were asked to have a chest X-ray. Our case finding identified TB disease in 13 of 97 people investigated, making these individuals a group worthy of attention.

Most studies have investigated health-seeking behaviour with respect to TB by interviewing TB patients. They have revealed patient knowledge issues and significant delays to diagnosis [10-12]. In this community-based study of coughing individuals, a key finding is that the majority did seek appropriate attention early. Is health system failure the main culprit in the delay to diagnosis and treatment that we observe? While the health system cannot be blamed for not performing sputum smear assessment in those with less than 3 weeks of cough (if they have no other key symptoms or signs) and this study did not ask whether such individuals were told to return if their cough persisted, it does appear that health system delay was important.

This study supports the findings of gender bias in passive case detection [13]: 9 (64%) of the cases identified were women, while approximately 70% of cases treated in the Farafenni area, and in The Gambia overall, are men [14]. Also, alternative case finding techniques may identify a sub-group of patients who are subject to increased delay. In the Gambian community there is a median delay from onset of the symptoms to treatment of approximately 8 weeks [15,16]. Median delays of up to 16 weeks have been reported elsewhere in Africa [17,18]. We observed even longer delays in the present study and in a previous alternative case finding study with traditional healers [19]. In the latter study we also found, consistent with the present study, that those visiting alternative health providers tend to be older than those who visit the health system.

In Kenya a single cough question first to household heads was the most successful method to identify TB cases in the community [20]. However, it is possible that we had a relatively low yield in The Gambia with this approach. In rural Uganda, 289 (5.2%) of 5574 people reported a cough of longer than 1 month's duration [21] Pronyk et a [22] identified 366 people with a chronic cough from a demographic surveillance population of 38,251. However, the yield from case finding was similar to our study: of 340 investigated, 6 sputum smear positive cases were identified.

Because case addresses are not routinely documented at the district hospital we do not know the number of sputum smear positive cases that were missed by our study. However, assuming approximately 30 cases per year are identified in the DSS population, with an average duration of cough (in those coughing for at least 3 weeks) of 4–9 weeks, one would expect to identify 1–4 smear positive cases in a cross-sectional survey such as ours. This is consistent with the 3 smear positive case cases that we found. Therefore, DSS patients diagnosed separately by the health system are likely to be small in number.

There has been considerable debate regarding the place of chest X-ray in the diagnosis of TB. Gothi et al [23] found in India, using sputum culture as the gold standard, that screening with X-ray at the population level offered little benefit over symptom screening. Harries et al [24] showed in Malawi, that when sputum culture is available, initial screening with chest X-ray followed by sputum analysis is not advantageous and may miss smear positive cases. However, sputum smear alone would have diagnosed less than half of those identified overall if chest X-ray had been offered to smear negative individuals. Furthermore, approximately one quarter of chest X-ray positive individuals would be expected to be negative on culture [24]. Therefore, in settings such as ours where sputum culture is not routinely available, it is advised to make use of the option of a chest X-ray if available and affordable. However, it is important that the X-rays are of good quality, that they are read by a radiologist or a physician experienced in X-ray diagnosis of TB, and only those considered to have an X-ray highly suggestive of TB are sent for treatment in the first instance [25].

## Conclusion

This study has identified possible ways to improve TB control in The Gambia. Firstly, the large majority of individuals who do seek appropriate help early need to be adequately investigated. Where chest X-ray is available and affordable (the cost is less than 2 Euro in The Gambia), especially in the absence of sputum culture facilities, it is recommended in conjunction with sputum smear. Secondly, educational efforts should encourage those with ongoing symptoms after an initial visit to the health system to seek help again. Community based case finding of itself may not offer added benefit: it is likely that the extra burden of disease in this study would have been identified through enhanced hospital case detection, since all the cases that were eventually diagnosed had sought help appropriately. However it may have a role in certain situations, such as where pockets of increased transmission are clearly identified. Enhanced case detection is a major priority for Global TB control. This study reinforces this urgent need and the importance of related health system delay [26,27]. While new diagnostic tools are required to realise this goal, some benefit can be gained in certain settings from the intelligent use of chest X-ray.

## Competing interests

The author(s) declare that they have no competing interests.

## Authors' contributions

YK was involved in the design of the study, supervised the field work and drafted the manuscript. MJ was involved in the design of the study, assisted in supervising the fieldwork and in the data analysis and write-up. TC was involved in the diagnosis of the TB cases by chest xray and was involved in the writeup. SD managed the data entry and verification and quality and contributed to the write-up. MA and RA supervised the microbiological aspects of the study and contributed to the write-up. AJ was involved in the design of the study, supervised the government diagnostic work and assisted in the writeup. PH conceived the study was involved in the design, analysis and write-up. All authors read and approved the final manuscript.

## Pre-publication history

The pre-publication history for this paper can be accessed here:



## Figures and Tables

**Table 1:** Characteristics of individuals at least 10 years old plus coughing for > = 3 weeks that were interviewed and the resident DSS population

**Characteristic**	**Coughing > = 3 weeks (n = 115)**	**DSS population (N = 29,871)**	**p value**
Residence, n(%)			
Village	55 (47.8)	11458 (38.4)	
Town	60 (52.2)	18413 (61.6)	0.034
Age, years			
Median (mean; range)	47 (46; 10–90)	25 (31; 10–104)	<0.001
Sex, n(%)			
Male	50 (43.5)	13596 (45.5)	
Female	65 (56.5)	16275 (54.5)	0.68
Ethnic Group, n(%)			
Wollof	28 (24.3)	11532 (38.6)	
Mandinka	50 (43.5)	9908 (33.2)	
Fula	33 (28.7)	6228 (20.1)	
Other	4 (3.5)	2203 (7.4)	<0.001

**Table 2:** Knowledge of tuberculosis in those coughing

**Knowledge question**	**Number (%)**
Heard of TB	91 (79.1)
Signs/Symptoms of TB	
Cough	53 (46.0)
Fever	10 (8.7)
Weight loss	24 (20.9)
Sweats	0
Cause of TB	
Don't know	87 (75.7)
Germ	11 (9.6)
God	11 (9.6)
Dirt	4(3.5)
Spirit	2 (1.7)
TB is transmissible	
Yes	92 (80.0)
No	2 (1.7)
Don't know	21 (18.3)
TB is curable	
Yes	93 (80.9)
No	3 (2.8)
Don't know	19 (6.5)

**Table 3:** Summary of TB cases identified

**Sex**	**Age (years)**	**Duration of cough (weeks)**	**Ethnic group**	**Smear**	**Chest X-ray findings**
M	14	3	Fula	Neg	Extensive patchy consolidation throughout the left lung field
F	40	104	Wolof	Neg	Bilateral patchy change, with focus of consolidation in the Right upper zone and probably effusion in the Left lower zone.
F	40	24	Mandinka	Pos	Not done-already on TB treatment when X-ray requested
F	40	572	Mandinka	Neg	Major shift of mediastinum to left with associated effusion and patchy change.
F	42	8	Wolof	Neg	Patchy consolidation throughout the left lung field, plus left upper zone cavitation and loss of lung volume.
F	50	156	Mandinka	Neg	Bilateral extensive patchy change with a small cavity and small pleural effusion on the left.
F	55	16	Mandinka	Pos	Left upper zone patchy change and cavitation.
M	60	104	Fula	Pos	Bilateral patchy change with cavitation and loss of volume in the right lung field.
M	60	23	Fula	Neg	Bilateral patchy change, with a focus of consolidation in the Right upper zone with associated loss of volume
F	60	4	Fula	Neg	Bilateral patchy changes, including loss of volume on the left.
F	62	9	Mandinka	Neg	Right upper zone patchy change and loss of volume
M	68	104	Mandinka	Neg	Right upper and mid zone consolidation and loss of volume.
F	70	12	Fula	Neg	Bilateral patchy change.
M	80	20	Mandinka	Neg	Right upper and mid-zone patchy change with loss of volume of that lung field.
